# Trends in the management of organic swine farm waste by composting: A systematic review

**DOI:** 10.1016/j.heliyon.2023.e18208

**Published:** 2023-07-14

**Authors:** Adriana Matiz-Villamil, Kelly Johana Méndez-Carranza, Andrés Felipe Pascagaza-Pulido, Tatiana Rendón-Rendón, Juliana Noriega-Noriega, Adriana Pulido-Villamarín

**Affiliations:** a Laboratorio de Biotecnología Aplicada, Grupo de Biotecnología Ambiental e Industrial (GBAI), Departamento de Microbiología, Facultad de Ciencias, Pontificia Universidad Javeriana, Bogotá, D.C., Colombia; b Unidad de Investigaciones Agropecuarias (UNIDIA), Departamento de Microbiología, Facultad de Ciencias, Pontificia Universidad Javeriana, Bogotá, D.C., Colombia

**Keywords:** Composting, Microbiological quality, Waste, Pig farm

## Abstract

Pig farming contributes to the economic development of nations and supplies human food demand; however, it generates a large amount of organic waste which, if not managed properly, becomes a risk to the environment and human and animal health. Considering the relevance of composting and its usefulness for the use of waste, this study aimed to determine the global trends in the management of composting manure, mortality and other organic waste produced on pig farms over the last five years (2017–2022). Systematic search involved four databases: ISI Web of Science, Scopus, Ebsco and Scielo. Of the total findings, 56 articles were included in the review, further classified into 14 categories for their respective analysis: co-substrates/additives, microbial communities, antibiotic resistance, heavy metals, polycyclic aromatic hydrocarbons, microbiological/parasitological quality, phytopathogens, nitrogen transformation, bioinoculants, comparison/combination with other waste management techniques, factors affecting composting, swine mortality and plant growth promotion/phytotoxicity. The review exemplified the importance of swine mortality composting as an alternative for organic matter management in pig farms, considering that the process also includes manure, vegetable waste and wood chips, among others. Controlled factors throughout the process are a requirement to obtain a stable product with physicochemical and microbiological quality that complies with national and international regulations and that will be useful and safe for application on crops, ensuring environmental, animal, and human health.

## 1 Introduction

Pig production has been consolidated to become one of the most important economic activities within the livestock sector in terms of obtaining animal protein for human consumption, reaching values of 107.65 million metric tons produced worldwide by 2021 [1,2]. Particularly in Colombia, pork production for 2021 was 491,233 tons, with Antioquia, Valle del Cauca and Bogotá D.C. being the leading departments in the country's pork industry [2].

Despite contributing to economic development and supplying food demand, pig farming generates a large volume of organic waste, including faecal matter, urine, wastewater from farm maintenance and cleaning, animal food waste and mortality including viscera, placenta, stillbirths, corpses and other body fragments, which, if not well managed, become a risk to the environmental, human, and animal health [3]. Environmentally, the impact of such waste affects the eutrophication of water bodies and the emission of greenhouse gases, along with other potentially polluting compounds that alter air quality [4]. In the field of human and animal health, particularly in backyard production systems where there is constant interspecies interaction between humans and domestic animals, the risk of transmission of zoonotic diseases increases if the waste produced in the pig production chain is not disposed of correctly [5].

Several porcine pathogens have been reported as zoonotic through animal production, causing zoonotic diseases. Although most diseases could be prevented through biosecurity and hygiene measures, such as hand washing, the most virulent strains of these microorganisms can cause serious illness. Some of the zoonotic infections transmitted through pigs are cysticercosis, tuberculosis, leptospirosis, influenza and hepatitis E (HEV), among others, through direct contact or consumption of food or water contaminated with faecal matter from infected pigs [[6], [7], [8]].

Pig manure contains urine, food waste, cleaning water and other farm waste; it is a good source of plant nutrients and can be used to improve soil fertility; however, when used fresh or untreated on crops, it can be a source of zoonotic pathogens that contaminate surface water and the general environment [9]. Improper waste management (collection, storage and disposal) can pose a health risk as manure may contain zoonotic pathogens capable of being transmitted through food and water intended for human consumption [10,11], among other transmission routes [6,8].

According to the report on the statistical portal “*statista”* in 2020, there were 677 million pigs worldwide, reflecting the continuous need for waste management [12]. The growth of the pig industry has led to the establishment of regulations requiring producers to ensure proper treatment and storage of manure and other organic wastes before the disposal or reuse of liquid or solid effluents [13]. Typical systems for manure management associated with pig production include some techniques that use liquid manure for storage in tanks for several months in the deep pit case and 2–8 weeks in the case of a pull plug. Other techniques use manure in solid forms, such as bedding of different materials for disposal and respective composting and solid-liquid separation in which the waste is entirely used [14].

On the other hand, sustainable alternatives exist, such as composting, defined according to the FAO as “*the mixing of decomposing organic matter under aerobic conditions used to improve soil structure and provide nutrients*” [15]. It is an aerobic process in which microorganisms transform organic material into more stable and useable compounds for soil and crops [16]. Composting brings several physical, chemical and microbiological soil benefits, such as improving soil structure for planting purposes, contributing to the distribution of nutrients [17], stabilisation and mineralisation of organic residues [16], production of humic substances [18], reduction of xenobiotic compounds such as pesticides [19], eradication of pathogens due to the high temperatures that the process undergoes (ensuring human health through stabilisation and treatment of zoonotic pathogens), odour control, recovery and prevention of soil desertification, the contribution of compounds such as nitrogen, potassium and phosphorus, and reduction of greenhouse gas emissions [17].

Composting as an alternative to organic waste management has not only shown to provide soil benefits, but has also been positioned as a sustainable activity within the framework of good practices for the proper management of environmental resources and the deceleration of climate change [20,21]. Indeed, this is directed to the achievement of the Sustainable Development Goals (SDGs) adopted by all United Nations Member States in 2015 as part of the 2030 Agenda for Sustainable Development [22], which aims to promote good health and well-being (3), access to clean water and adequate sanitation (6) and responsible production and consumption of resources (12) through composting, by reducing water pollution and the proportion of untreated wastewater, increasing the safe management of resources and reducing health risks associated with exposure to pathogens [23]. Likewise, composting strategies also contribute to the development of more sustainable cities and communities (11), and climate action (13) by decreasing the production of different types of waste that were neither recycled nor reused, as well as the reduction of greenhouse gas emissions as a product of poor waste management [21].

During the composting process, it is possible to identify three phases according to temperature: mesophilic, thermophilic and maturation phase (also mesophilic). In addition, to obtain a stable product, it is necessary to consider certain fundamental factors such as pH, temperature, aeration/oxygen, moisture and carbon/nitrogen (C/N) ratio, among others [24]. Considering the importance that composting has in waste utilization, this study aimed to determine worldwide trends in manure composting management, mortality and other organic waste produced on pig farms over the last five years (2017–2022).

## 2 Materials and methods

Systematic search involved four databases: ISI Web of Science, Scopus, Ebsco and Scielo; using as search equation for the fields Title-Abstract-Keywords: (compost* AND (“swine mortality*" OR pig) AND (manure OR household* OR “household waste")) and taking into account articles published between 2017 and 2022. We filtered the information by document type, considering only original papers in English, Spanish and Portuguese. There was no geographical delimitation of the filtered articles, neither was made a comparison between countries. Subsequently, the eight criteria listed in Table 1 allowed us to evaluate the article's selection. After assigning each parameter a quality score (using a scale from 0 to 11), those articles with a final score ≥ of 8 were for systematic review. The following exclusion parameters allowed for generating the list of articles for the systematic review:

● Articles related to the application of compost on crops and not only to the production of compost.
● Pig mortality and/or pig manure not included in the raw materials to be processed.
● Composting in systems other than windrows or bins.

**Table 1:** Applied criteria to the shortlisted articles to assess their eligibility.

Criteria	Description	Criteria level
1	Specific data on the type of waste that was used in the process	(0) Not included (1) Partially included (2) Fully included
2	Physico-chemical and microbiological characterisation of raw materials and the final product	(0) Not included (1) Partially included (2) Fully included
3	Combined composting of pig mortality, pig manure and/or household waste	(0) Not included (1) Partially included (2) Fully included
4	Windrow and/or bin composting	(0) Not included (1) Included
5	Microbiological quality of the final product - absence of pathogens	(0) Not included (1) Included
6	Automated composting systems	(0) Included (1) Not included
7	Composting of other materials such as plastic	(0) Included (1) Not included
8	Anaerobic composting systems	(0) Included (1) Not included

## 3 Results

**Fig. 1:**
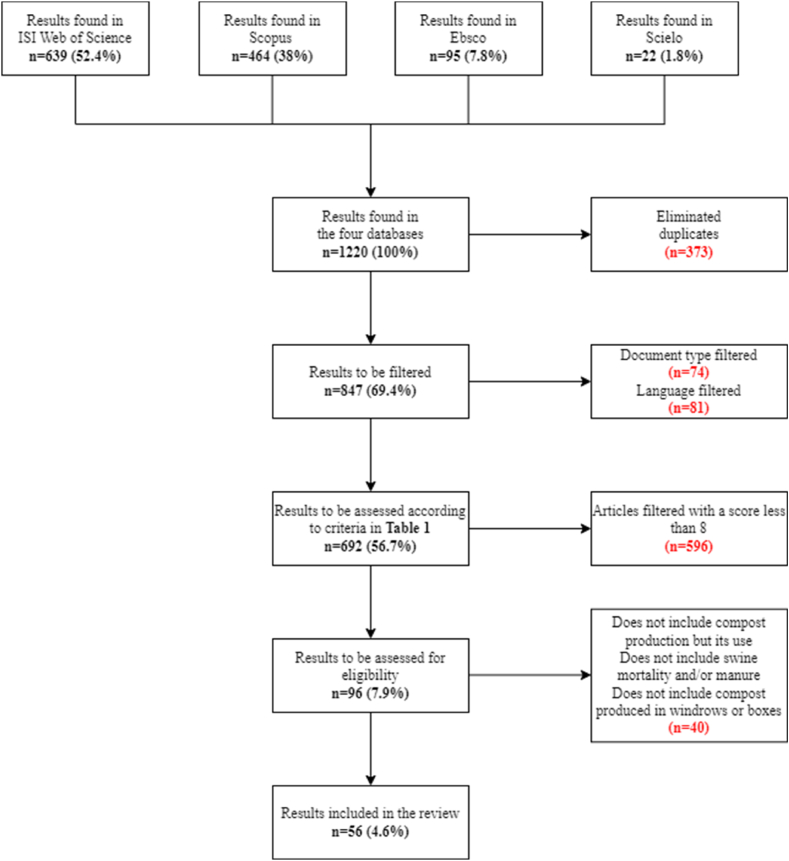
Search flow and selection of articles for systematic review.

1220 (100%) results were preliminarily found in ISI Web of Science, Scopus, Ebsco and Scielo (Fig. 1), and then reduced to 847 documents after eliminating duplicates. After selecting the original articles in English, Spanish and Portuguese, 692 preliminary results remain. Then the evaluation process of eight listed criteria (Table 1); subsequently, results in 96 documents that, taking into account the exclusion criteria, were limited to 56 articles as the total number included in the systematic review and classified in turn into 14 categories for their respective analysis (Table 2).

**Table 2:** Categories for the classification of articles included in the systematic review and the number of articles per category. It is important to note that some categories appeared in the same document, which is why the sum of the items is greater than the 56 selected items.

Category	Number of items included (%/108)
Cosubstrates/additives	23 (21.3)
Microbial communities	20 (18.5)
Antibiotic resistance	9 (8.3)
Heavy metals	7 (6.5)
Polycyclic aromatic hydrocarbons	1 (0.9)
Microbiological quality	7 (6.5)
Parasitology	3 (2.8)
Phytopathogens	1 (0.9)
Nitrogen transformation	10 (9.3)
Bioinoculants	10 (9.3)
Comparison/combination with other waste management techniques	6 (5.6)
Factors affecting composting	5 (4.6)
Pig mortality	4 (3.7)
Plant growth promotion/phytotoxicity	2 (1.9)

## 4 Discussion

### 4.1 Co-substrates, support materials and other additives

As part of the composting process, materials, most of plant origin, are incorporated for a wide variety of purposes, such as adjusting the carbon-to-nitrogen (C/N) ratio in the system, moisture content and porosity, as well as improving oxygen diffusion and thereby stimulating microbial metabolic activity [25,26]. Some of the most frequently used materials were: sawdust and rice or wheat straw; however, the raw materials considered in studies in recent years have diversified. Wong et al. [27], implemented paper and wood chips as co-substrates for swine manure composting in a 3:2:1 ratio. The use of materials of forestry origins, such as brush litter, chestnut fruit shells and medium-density fibre panels made from pine residues, have resulted in a quality product when used as co-substrates for swine manure composting [28]. Cultured residues of macromycetes, such as *Auricularia auricula* and *Flammulina velutipes* are also effective co-substrates for swine manure compost production [[29], [30], [31]].

Another purpose for which co-substrates of different types serves is to prolong the thermophilic phase; Liu et al. [32] and Tu et al. [33], obtained such an effect after implementing the bamboo biochar addition to the composting of pig manure. Similarly, Gao et al. [34] found that composting the same manure mixed with human faecal matter, kitchen waste and rice straw resulted in a temperature increase exceeding 50 °C.

Regarding the effect of different co-substrates on the nutritional composition of the final product, studies have focused on reducing nutrient loss, specifically on carbon and nitrogen (considered a limiting factor in compost production) [35]. Yang et al. [36] found that 10% (w/w) bean hull incorporation into swine manure compost reduces N_2_O emissions. Ren et al. [37], attributed the reduction in CH_4_ and N_2_O emissions to the adsorptive properties of clay. Other inorganic compounds used include fertiliser components such as calcium superphosphate, which in addition to decreasing CO_2_ emission, retains nitrogen in the system and increases the concentration of humic substances [38,39]. Waste transformed by physical treatment also served as co-substrates for gas emission reduction. In addition to increasing compost maturity, 10% coal gasification residues limit CH_4_ and N_2_O emissions [40]. Biochar produced from macromycetes residues and corn stalks and stalks decrease CO_2_ emission and organic matter loss as NH_3_ [41,42].

The addition of materials for reducing the bioavailability of heavy metals involved different mechanisms. Yang et al. [43] reported that sponge and cotton directly increase the cooper adsorption, cadmium and chromium. Copper precipitation mediated by inorganic compounds such as rock phosphate and boron residues, was previously described by Wang et al. [44]. An additional mechanism corresponds to the formation of complexes with humic acids produced during the transformation of organic matter and which are associated with copper and zinc when incorporating substrates such as corn straw, peanut shells and sawdust [45,46].

Regarding the effect of substrate on the presence of antibiotic resistance genes in pig manure compost, 10% coal gasification residues and corncob stover decrease the abundance and diversity of antibiotic resistance genes. The authors explain that the high carbon-to-nitrogen (C/N) ratio in the case of corncob stover and the porosity of coal gasification residues support a wide variety of microbial communities that contribute to the attenuation of antibiotic resistance genes in the final product [47,48].

Another effect of co-substrates addition to pig manure as compost feedstock is the modification of microbial communities. Zhu et al. [49] reported that reed straw in processing with nitrogen-rich materials, including pig manure, promotes the complexity of thermophilic microbial communities. The addition of pine leaf biochar strengthened fungal diversity when incorporated at a concentration of 10% (w/w), according to Li et al. [50].

### 4.2 Microbial communities

Composting is carried out by microorganisms, either from the incorporated raw materials or from bioinoculants; therefore, understanding the structure and function of the microbial communities is crucial to get high-quality products [31]. Generally, the bacterial phyla reported in different studies include Actinobacteria, Bacteroidetes, Chloroflexi, Firmicutes and Proteobacteria, acting at diverse stages of the process. However, when comparing the composting of different types of manure, the phylum Firmicutes is defined as the dominant one, specifically in the treatment of swine manure, due to the formation of endospores at high temperatures such as those reached in the thermophilic phase and its ability to degrade a wide variety of starches and sugars [47,51,52].

Authors have studied the effect of the addition of different substrates on the structure and functionality of microbial communities. Yang et al. [36] reported increased bacterial richness and diversity by including wheat straw and bean husk during swine manure composting, highlighting the role of the Bacilli class throughout the process. The addition of reed straw results in the formation of thermophilic bacterial communities, mainly belonging to the phyla Actinobacteria and Firmicutes, involving genera such as Thermopolyspora, Thermobispora and Rhodothermus; moreover, in terms of fungal diversity, representatives of the phylum Ascomycota such as Thermomyces, Talaromyces, Aspergillus and Wallemia were reported [53]. Wang et al. [54] isolated Thermomyces and Aspergillus, highlighting their role in the decomposition of organic matter due to the enzyme battery they possess. By including culture residues of macromycetes, such as *Auricularia auricula*, the nitrogen transformation affects the composition of microbial communities; additionally, Pseudomonas is a relevant bacterial genus due to its plant growth-promoting activity [30,55].

Other substrates such as pine leaf biochar generate changes in fungal composition depending on the dose added, with some fungal genera such as Heterobasidion, Pezoloma, Mucor, Geastrum, Talaromyces and Cystofilobasidium being dominant [50]. Ma et al. [56] reported the predominance of bacteria of the genera Bacillus, Luteimonas, Clostridium, Pseudomonas and Streptomyces when adding sugarcane bagasse to swine manure compost. The addition of 7.5% (w/w) coconut husk biochar not only increases the bacterial diversity but also increases the degradation of keratin in pig hairs by Bacillus [57]. Wang et al. [58] described the activation of metabolic pathways associated with the degradation of xenobiotics, also, the increase in the diversity of bacterial communities by including pumice stone as a substrate for pig manure composting.

Understanding microbial communities has been essential to addressing the presence of antibiotics and heavy metals in pig manure. Ma et al. [59] reported reduced bacterial diversity in swine manure compost from pigs fed chlortetracycline, which affects pig growth by decreasing the abundance of genera such as Prevotella, whose metabolism contributes to the immune response against pathogens. Some bacteria carrying antibiotic-resistance genes have been from the phyla Actinobacteria and Firmicutes and within the genre Ruminofilibacter, Luteimonas and Pseudodidiomarina [47,48,60]. The effect on bacterial communities is reflected in their diversity and abundance and their role within the composting process when affecting nitrogen transformation and the maturation process of organic matter [61]. Liu et al. [61] contaminated swine manure with amoxicillin to inhibit ammonia-oxidising and ammonium-oxidising bacteria and to significantly increase the denitrifying bacteria leading to an increase in nitrogen loss by denitrification.

Related to the behaviour of microbial communities during the treatment of pig manure contaminated with heavy metals, a decrease in bacterial richness and abundance has been observed in the presence of elements such as arsenic (112.5 mg/kg de Na_3_AsO_4_ 12H_2_O) and copper (662.5 mg/kg CuSO_4_ 5H_2_O) [62]. Yang et al. [43] incorporated different support materials (sponge, cotton and zeolite) for the adsorption of zinc, lead, copper, nickel and cadmium to increase the abundance of functional bacteria for the composting process, whereby the bacterial diversity of the system was reduced (Shannon-Wiener index between 2.0 and 2.5) compared to the control pile (2.70–2.92).

The addition of microbial inoculants also affects the microbial communities present in the composting system, which varies according to the composition of the bioinoculant. When inoculating *Streptomyces griseorubens* JSD-1, the bacterial diversity decreases with a Simpson's index of 0.8 compared to the control value (0.9) because the actinobacteria produces secondary metabolites that either compete for or modify the nutrients in the system, thus shaping the structure of the bacterial communities [51]. Li et al. [63], reported an increase in bacterial diversity (Shannon index of 4.5 and 6.0 before and after the thermophilic phase, respectively) when inoculating *Acinetobacter pittii*, *Bacillus subtilis* sub sp. *stercoris* and *Bacillus altitudinis*, as they rapidly degrade organic matter, increasing the availability of nutrients and thus the metabolism of other bacteria present in the raw materials. Yang et al. [35], described a similar effect when inoculating bacteria involved in the nitrogen cycle (*Bacillus cereus*, *Pseudomonas donghuensis*, *Bacillus licheniformis*, *Klebsiella pneumoniae* and *Pseudomonas aeruginosa*) during the composting of swine mortality with wood chips. Authors observed an increase in the richness and diversity of other microbial communities at the end of the process (the chao1 index increased from 1. 000 to 1,500, as the Shannon index did from 6 to 8) as a consequence of microbial diversity activity in nitrogen transformation.

### 4.3 Heavy metals, antibiotic resistance and polycyclic aromatic hydrocarbons

The abundance of heavy metals as environmental pollutants poses a risk to different organisms due to their high toxicity and persistence [43]. Among these metals, copper (Cu) and zinc (Zn) are the most studied due to sustainable alternatives, such as composting, to reduce their content. Pig manure composting and pyrolysis (to obtain biochar) are two alternatives of those evaluated by Meng et al. [64], where composting reduced the bioavailability of both metals (from 115.11 to 59.99 to 31.37 and 21.95 mg/kg, respectively) with a clear difference between their concentrations before and after the 84 days process. Furthermore, the chemical speciation of the metals was different, as copper remained bound to the organic fraction, while zinc was associated with the reducible iron-manganese bound fractions.

The use of biochar during the composting process is of great utility, according to Li et al. [45], because it was possible to immobilise copper (70.36%) in one of the treatments (not specified by the authors) as well as zinc (40.76%) in another; this may be related to the formation of fulvic and humic acids that promote the transformation of heavy metals to forms with more stability. Likewise, immobilisation may also be associated with the C/N ratio employed during the composting process, as verified by Wu et al. [46], using a C/N of 25, which allowed the immobilisation of both metals with 90% of copper belonging to the residual fraction in the final product. However, the copper shows higher susceptibility to additives (such as rock phosphate and boron residues), compared to zinc, through a decrease in its bioavailability factor (found in oxidisable and residual fractions) [44]. The authors attribute this effect to the additives used and their possible decrease in the concentration of fulvic acids, as the difference in the ratio between fulvic and humic acids may be associated with changes in the bioavailability of copper. Concerning zinc, its source is solid pig manure since when using the liquid fraction, there are lower concentrations of this metal which, with the appropriate co-substrates, can be reduced [65].

Additionally, the possibility of immobilising metalloids such as arsenic (As) and degrading antibiotics such as oxytetracycline is also demonstrated by composting [62]. Similarly, there are studies involving other metals such as lead (Pb), chromium (Cr), nickel (Ni) and cadmium (Cd), which evaluate the use of bacteria and absorbent materials during the composting process, most notably the use of cotton wool and sponges [ 43].

On the other hand, the liquid residue of oils is another pollutant to consider, as it can have cytotoxic, mutagenic and carcinogenic effects. It is usually composed of volatile and semi-volatile organic compounds, such as polycyclic aromatic hydrocarbons. These compounds tend to contaminate the soil and then generate pressure to find microorganisms capable of degrading them. Among them, some *Bacillus* sp., *Arthrobacter* sp., and *Staphylococcus* sp., were isolated and identified after a mixed composting process between contaminated soil and manure from different animals (pigs, cattle, horses, poultry), where a reduction (77–99%) in the concentration of polycyclic aromatic hydrocarbons was found [66].

Although the use of waste is fundamental for environmental health, most consider that raw materials can be carriers of abundant antimicrobial resistance genes, and it may become a transmission pathway through the final product that eventually generates contamination in the food chain and affects human health if composting has not been properly done [60]. Several studies have been carried out with different antibiotics to evaluate the efficacy of composting for the removal of resistance genes (ARGs) and mobile genetic elements (MGEs).

The research by Ma et al. [59], compared composting of pig manure by anaerobic digestion with the possibility to determine that composting was more efficient in reducing the relative abundance of resistance genes such as *tetC, tetQ, tetW, intl1* and *intl2* by 96.37, 98.63, 92.48, 79.45 and 96.84%, respectively. Have been proved that, in the thermophilic phase (temperatures between 44 and 65 °C), more than 90% of the *mcr*-1 gene, which confers resistance to colistin in enterobacteria, was removed and that this gene came from the original raw material [67]; this has also demonstrated in other studies [56]. Cheng et al. [68], showed that the removal efficiency of antibiotics (oxytetracycline, sulfamerazine, ciprofloxacin) was higher when supplying the antibiotics as fed for pigs than when added directly to the manure. The addition of antibiotics, such as amoxicillin, can be detrimental to the process by slowing maturation and affecting the structure and succession of bacterial communities [61]. Shehata et al. [62] evidenced that microbial communities adapt to the environment when evaluating a combined treatment between antibiotics (in this case, oxytetracycline), heavy metals and metalloids, where they maintained their diversity and abundance until cooling.

The addition of substances to the process may also play a role in reducing the presence of resistance genes. Composting swine manure with corn stover or “cob” (residues produced after shelling the corn cob) [48], charcoal residues [47] and green tea residues [60], has shown effective removal of resistance genes (ARGs) and mobile genetic elements (MGEs) (in the range of 5.1–95.9% for Lu et al. [47]). This means that composting process is of high usefulness even on recalcitrant compounds due to the microbial action.

### 4.4 Microbiological quality

When using pig manure as a raw material to obtain a product for agricultural use, it is necessary to consider its origin and its capacity to be a substrate for the development of parasites and other pathogens; however, there are few studies aimed at ensuring the safety of compost [69]. Wong et al. [27], reported the reduction of pathogenic bacteria such as *E. coli*, Salmonella and faecal coliforms during swine manure's compost with wood chips. The use of black soldier fly larvae (*Hermetia illucens*) as a pre-treatment of swine manure contributes to the decrease of pathogens belonging to the *Proteobacteria phylum* (*E. coli*, Salmonella, Vibrio) by 30% compared to the untreated control (80% abundance) [70]. In contrast, despite achieving thermophilicity, Arias et al. [28] did not eliminate pathogens (*Salmonella* spp. and *E. coli*), a result they attribute to the ability of these microorganisms.

Regarding the detection of parasites, Matiz-Villamil et al. [71] reported in pig manure the presence of protozoa such as Cryptosporidium, Eimeria and *Balantidium coli* with eggs of nematodes of the genera Strongylus and Trichostrongylus, bacteria such as Clostridium and coliforms such as Salmonella. After the composting, there was a reduction of pathogens and parasites, in addition to the control of arthropod pests due to the incorporation of entomopathogenic nematodes, so that the final product complied with the Colombian regulations (NTC 5167-2011), which require the absence of *Salmonella* spp. in 25 g of the final product and a count of enterobacteria <1000 CFU/g final product; thus, constituting an alternative for the proper management of swine mortality. The study by Ramires et al. [72], also reported the presence of protozoa, such as coccidial oocysts and *Entamoeba cysts*, in swine manure. On the other hand, concerning the methodologies for the detection of helminth eggs, De Sá et al. [69] established that the modified Bailenger method (concentration technique with acetic acid, ether and zinc sulphate) allows the recovery of *Ascaris suum* eggs in liquid swine manure samples to optimise the detection of this parasite and not to underestimate the importance of its study in the production of this type of manure.

Regarding plant pathogens, little research has considered their identification in both raw materials and the final product. Meng et al. [29] found a reduction in Fusarium copy number/μL of up to 50-fold compared to the other evaluated treatments by mixing compost from pig manure, biogas production residues and *Auricularia auricula* macromycete cultures, together with peat and perlite in a 4:1:1 ratio. Likewise, inoculation of *Trichoderma atroviride* with biocontrol potential of the genus Trichoderma exhibited a decrease of plant pathogens such as Penicillium (36%), Rhizopus (7%), Alternaria (4%) and Mucor (5%) [73].

### 4.5 Nitrogen transformation

Nitrogen is the nutrient that constitutes the major limiting factor for final product use due to its loss in the form of gases that, when volatilised, pollute the environment [35]. That is why analysis of nitrogen behaviour during the swine manure composting process is relevant. According to Yang et al. [35], ammonification occurs during the early stages of the process, while nitrification, denitrification and nitrogen fixation occur in the later stages. In this study, the inoculant extended the thermophilic phase and was in these treatments where a total nitrogen content of up to 50% higher, compared to the control (not inoculated), was found. The addition of different substances (e.g. bioinoculants) and their relationship with a prolonged thermophilic phase (11–13 days above 55 °C) was also present in the research of Yang et al. [36], where co-substrates such as bean husks was used. However, the co-substrate also influenced different stages of the process, increasing NH_3_ (ammonia) emission and, in turn, decreasing N_2_O (nitrous oxide) emission, implying a decrease in nitrogen loss, although it was associated with the co-substrate proportion. The same effect on the thermophilic phase and nitrogen loss may occur when using clay [37] and a fermentation agent [74], while acid substrates (such as phosphates and rotten apples) [38] and the use of the liquid fraction of pig manure [65], only affected nitrogen loss.

Another aspect that influences nitrogen activity is how the composting process is made, e.g.g, when it is employed in an ectopic fermentation bed system, with a constant flow of this nutrient, NH_3_ emission decreases and microbial communities capable of predominantly performing nitrification and denitrification processes are found [55]. However, factors such as the use of antibiotics (amoxicillin) affect the communities, causing a loss of nitrogen through denitrification [61]; similarly, performing the composting process in cold seasons (<10 °C) generated a higher emission of N_2_O which also indicates nitrogen loss [75].

Finally, for the measurement of nitrogen content, direct methods, such as spectrophotometry on nitrate reductase enzyme [55] or colourimetry to determine urease activity, were frequently used [76].

### 4.6 Bioinoculants

There is a wide variety of bioinoculants, composed of different genera of bacteria and fungi, with diverse effects on composting. One of the aspects to consider when using bioinoculants is their ability to counteract the effects of other microorganisms found, either in the soil or in the final product. Wolna-Maruwka et al. [73], evaluated the antagonism effect of different treatments with a *Trichoderma atroviride* and two strains of *Trichoderma harzianum* on microorganisms commonly found in the composting of pig manure with onion and tomato residues. The different Trichoderma strains were antagonistic agents against phytopathogenic microorganisms such as Penicillium, Mucor, Rhizopus and Alternaria, whose abundance reduced in the final product with percentages ranging from 4 to 36%.

Chi et al. [51] used *Streptomyces griseorubens* strain JSD-1 in pig manure composting. Among the effects of the inoculation of this actinobacterium, a thermophilic phase was reached quickly, with a longer duration and an improvement in its nutrient content, specifically nitrogen, phosphorus and potassium. Similar effects occurred in the study by Zhu et al. [53], which used strains of *Gloeophyllum trabeum* and concluded that, after inoculation, the ripening process accelerates and the degradation of lignocellulosic components improved due to the increased activity of xylanases, manganese peroxidases and laccases.

Regarding the effect of bioinoculants on nitrogen transformation during the composting process, Tu et al. [33] obtained an increase in total product nitrogen by adding biochar and inoculating with Lactobacillus, Flavobacterium, Candida, Bacillus, Actinomadura, Solibacillus and Psychrobacter (59%), compared to the control (31.8%); which is related to the porosity of biochar in adsorbing nitrogenous compounds. Even without biochar, the mineralisation of organic forms of nitrogen to available forms is due to bioinoculants use [74].

Yang et al. [35] evaluated nitrogen-transforming strains and found that they degrade pig mortality tissue in a shorter time, thus prolonging the thermophilic phase of composting; in addition, the final product showed an increase in the availability of this nutrient. In turn, in the study by Matiz-Villamil et al. [71], a bioinoculant consisting of strains of *Bacillus subtilis*, *Talaromyces sayulitensis* (HC1), *Steinernema* sp., and *Heterorhabditis* sp., was used, which allowed an advance in the maturity of the swine mortality compost, reduced the appearance of pests such as flies around the assembly and obtained a microbiological quality following the regulations (NTC 5167-2011). On the other hand, Vargas et al. [77] evaluated an endogenous inoculum; in which *Bacillus* sp. was isolated and reduced in the concentration of *E. coli* at the end of the process (0 Log_10_ compared to 8.52 Log_10_ obtained at the beginning of the process).

Another effect of bioinoculant use is related to changes in microbial communities. Li et al. [45], reported an increase in the abundance of cellulose-, hemicellulose- and lignin-degrading bacteria of the genera Flavobacterium and Solibacillus when inoculated *Acinetobacter pittii*, *Bacillus subtilis* subsp. *stercoris* and *Bacillus altitudinis*.

Finally, there is also interest in the influence of bioinoculant use on composting processes aimed at heavy metals reduction or removal. Li et al. [63] have proved the immobilisation capacity of copper and zinc by adding biochar from sawdust, corn straw, peanut shells and a bioinoculant composed of lactic acid bacteria, yeasts, *Bacillus* sp., photosynthetic bacteria and actinomycetes.

### 4.7 Combination and or comparison with other waste management techniques

In general, several biological and physical strategies allow the management and utilization of waste, such as farm animal manure [64]. Particularly for pig manure management, studies have combined composting with other techniques. Pretreatment with larvae of the black soldier flies *Hermetia illucens* has proven beneficial not only by concentrating the production of humic substances and available organic matter but also by decreasing pathogenic bacteria present in the final product, such as *E. coli*, *S. aureus*, Salmonella, Enterococcus, Vibrio and Bacillus [70,78]. On the other hand, Galliou et al. [79] implemented solarisation of wastewater from olive oil production to be used as a compost feedstock with pig manure and grape pomace, obtaining a nutrient-rich product and applying it to paprika crops.

Studies have also compared swine manure composting with other waste management techniques. Ma et al. [59], investigated the attenuation of chlortetracycline by anaerobic digestion and composting, the latter being the most efficient treatment not only in reducing the concentration of the antibiotic but also the abundance of antibiotic resistance genes and mobile genetic elements. However, composting is not always the best alternative; compared to pyrolysis for obtaining biochar from swine manure and sawdust because copper and zinc concentrations decreased to a lesser extent by composting [64]. When contrasting the effect of compost and swine manure vermicompost on morphological variables of lettuce, radish and rice seeds and seedlings, the compost had a phytotoxic effect on these plant species due to low pH values and high electrical conductivity compared to vermicompost, which exhibited a positive effect on the plants studied [72].

### 4.8 Factors affecting the composting process

As composting is a biological process, microorganisms play a crucial role in its development and must be considered throughout the treatment the factors affecting their growth and reproduction. Among the most important factors to consider are aeration, temperature, pH, humidity, substrate type, size and raw material proportions (C/N ratio), which are continuously monitored to ensure they are within the required ranges with the aim that the process will be carried out properly [80].

Arias et al. [28] identified that the particle size of the raw materials and the degree of compaction of the compost pile affect the ability to reach thermophilic temperatures and their maintenance over time; this is because a high compaction degree of the composted materials leads to an increase in bulk density. Consequently, the free air spaces, which maintain the compost pile with a higher aeration rate, are reduced, so reducing them allows heat to keep conserved within the composting matrix [81], especially in the forest biomass used in the study. Fan et al. [82] observed that the use of rice husks in small sizes (1 mm) for the composting of liquid swine manure increased the efficiency of the process by prolonging the duration of the thermophilic phase (10 days, compared to 7 mm fragments whose thermophilic phase duration was 8 days) and also reduced the generation of leachate (0.03–0.1 L/m^3^ day compared to other studies with values of 0.63–1.39 L/m^3^ day).

Concerning the porosity of raw materials, Ren et al. [37] evaluated the effect of a mineral additive (clay) on greenhouse gas (GHG) emissions. They found that by adding it to a mixture of pig manure and sawdust for the production of compost, they managed to reduce CH_4_ and N_2_O emissions by 45.88 and 86.79%, respectively; this is because clay, being a porous mineral, promoted oxygen diffusion within the compost mass, thus decreasing anaerobic conditions, which limits the activity of methanogenic microorganisms [83] and nitrifying bacteria [84].

Temperature is an indicator that reflects the metabolism of microorganisms in composting mixtures, but, at the same time, it affects their activity and, therefore, the process. Arias et al. [28] showed the necessity to reach temperatures above 40 °C and also to maintain them over time, considering that the elimination of pathogenic microorganisms or the reduction of their microbial load (in the case of the study, *Salmonella* spp. and *Escherichia coli* found in substrates such as pig manure) is necessary. A similar result occurred in two of the four treatments of the research because the thermophilic phase in these had a duration of more than 20 days. Likewise, Ren et al. [37] found that the use of clay prolongs the thermophilic phase of composting, which is concerned with the fact that the addition of the porous mineral allows more microorganisms to degrade organic matter, evident in the production of more heat as a result of the release of energy, a product of microbial metabolism. In this way, by having more material degradation, the clay allowed a C/N ratio (18.35) decrease compared with the control (21.65), indicating the maturity of the compost and the promotion of the formation of humic substances.

The limited oxygen supply to the composting system (whose value should not be less than 5% and at its optimal level should be 10%) decreases the temperature necessary for the process. Therefore, the microbial activity [80], favours anaerobic conditions, allowing a higher production of GHG, slowing down the process and resulting in a lower degradation of organic matter [85]. Consequently, external aeration affects the thermophilic temperature range and the degradation time of organic substrates. According to the findings of Jiang-Ming [85] in the production of compost from pig manure and Enoki macromycete crop residues, there is evidence of an inversely proportional behaviour between the duration of the thermophilic phase, the reach of higher temperatures and the periodicity of turning, which can be associated with the promotion of aerobic microbial activity by the constant supply of oxygen, allowing a stable product obtained in a shorter period. The author also describes a direct and proportional relationship between the frequency of turning and moisture loss in the swine manure composting system: turning every other day resulted in a product with moisture of about 30% due to the volatilisation of water due to higher temperatures and aeration. Arias et al. [28], described a similar effect and indicated that the early development of thermophilic temperatures resulted in a sudden decrease in moisture content, compensated by additional amounts of liquid swine manure.

An additional factor to consider when evaluating the quality of the composting process is salinity since it is one of the limitations of its application, given that plants exhibit low tolerance to saline conditions. For this reason, Bustamante et al. [86], evaluated the effect of washing the pig manure compost with sterile distilled water before its application on paprika seedlings, finding that in addition to reducing the salinity of the product without affecting its nutritional composition, it had a positive effect on the germination and growth of the plant species studied.

### 4.9 Pig mortality

Looking at the global increase in pig production is necessary to find an alternative to mortality use because traditional methods are environmentally unsafe [71]**.** One such option is the composting of mortality which, in studies by Vargas-Sanchez et al. [77]and Matiz-Villamil et al. [71], demonstrated the use of bioinoculants to accelerate the decomposition of carcasses and reach product maturity in less time. Furthermore, the addition of bacteria able of nitrogen conversion also contributes significantly to the increase of total nitrogen content [35].

In some cases, considering that mortality is part of the raw materials involved in the process, it has been proven that the risk of transmission of pathogens present in carcasses (*Salmonella* sp., vaccine strains of Bovine herpesvirus-1 (BHV-1) and Bovine Viral Diarrhoea Virus (BVDV)) is minimal in composting systems with plastic wrapping [87].

### 4.10 Plant growth promotion and/or phytotoxicity

Compost is a source of macro-, micronutrients, organic matter and compounds that support and promote plant growth [16]. However, when compost is unstable, has low nitrogen levels, low/high pH values or high salt concentrations, these parameters can interfere negatively with plant development due to insufficient nutrients or the possible presence of phytotoxic compounds. Consequently, laboratory tests evaluate the final product's plant growth promotion effect and phytotoxicity [88].

In the study conducted by Ramires et al. [72], the compost obtained from pig slaughter plant waste had a phytotoxic effect on germination, root length and aerial development of lettuce (*L. sativa* L.), radish (*R. sativus* L.) and rice (*O. sativa* L.) plants. Its application on seeds of each plant species after 365 days of stabilisation of the composted material showed a germination inhibition percentage of 90% for lettuce, 88% for radish and 57% for rice, as well as the lowest values for root length, aerial development, fresh and dry weight, with significant differences compared to the other treatments, which may be related to the production of toxic compounds during processing and high values of electrical conductivity. The authors found that the value of electrical conductivity, being an indicator of the concentration of ionised salts in the sample and representing a parameter for estimating the salinity of the materials and the osmotic potential, may be related to damage to the plasma membrane of the seeds due to a high concentration of salts in the compost, which caused seed salt stress.

In contrast to the findings of Ramires et al. [72] and Meng et al. [64] showed that compost derived from pig manure, biogas production residues and substrate degraded by *A. auricula* fungi, as a partial or total replacement of peat in a medium for growing tomato and paprika seedlings, increased pH values, electrical conductivity, porosity, bulk density and NPK levels. The application of the compost did not affect the germination percentage of tomato and paprika seeds and also resulted in higher or comparable values with those obtained in the controls for the evaluated plant growth parameters (number of leaves, aerial length, stem diameter, leaf area, leaf chlorophyll content, fresh weight and dry weight of the seedling), which is attributed to the fact that the nutrients necessary for plant growth are available in the compost, especially nitrogen and potassium. This allowed the authors to determine in the study that the use of compost produced from the above-mentioned materials is a good alternative to the use of peat.

When compared to other methods, some additional benefits of composting besides the several approaches analyzed above include its low cost, the production of nutritionally valuable compounds at the end of the process and the improvement of soil physicochemical and microbiological properties. Despite a long time until the obtaining of the product, the presence of some thermotolerant pathogens and their reactivation, composting stands out as one of the most effective alternatives regarding waste management [89,90].

## 5 Conclusion

In conclusion, the review exemplifies the importance of manure and or swine mortality composting as an alternative capable of ensuring environmental, animal and human health, whose process must have controlled factors to obtain a stable product with adequate microbiological quality, which is useful and safe for application on crops, reducing the risk of aggravating problems such as antimicrobial resistance. It is pertinent to mention that composting can be carried out with or without bioinoculants or co-substrates, but their use during the run time has shown considerable benefits. On the other hand, it can contribute to other processes, such as the removal of heavy metals and polycyclic hydrocarbons, which gives value to its application in different contexts and highlights its relevance as an alternative for the treatment of waste-containing compounds difficult to degrade.

## Funding and acknowledgements

This work was financed by “10.13039/501100006280Ministerio de Ciencia y Tecnología” and “10.13039/501100009543Pontificia Universidad Javeriana” Bogotá, D.C. Colombia; as part of a call for applications 874–2020, Grant ID: 09336, titled “Desarrollo de una estrategia de compostaje a partir de residuos domiciliarios y de grajas porcícolas en los corregimientos Zabaletas y Santa Rosa”.

Authors, thanks to Dr., Raúl A. Poutou-Piñales, Ph.D., for English Editing.

## Author contributions

**A.M.-V**. Investigation, conceptualization, supervision, methodology, writing review and editing. **K.J.M.-C**. Methodology, formal analysis, and writing the original draft. **A.F.P.-P.** Methodology, formal analysis, and writing the original draft. **T.R.-R.** Methodology, formal analysis, and writing the original draft. **J.N.-N**. Methodology, formal analysis, and writing the original draft. **A.P.-V**. The investigation, project administration, conceptualization, writing review and editing.

## Declaration of competing interest

The authors declare that they have no known competing financial interests or personal relationships that could have appeared to influence the work reported in this paper.
