# Supplementary material for: Trends in the management of organic swine farm waste by composting: A systematic review
Source: Heliyon. 2023 Jul 14;9(8):e18208. doi: 10.1016/j.heliyon.2023.e18208 (PMC10412907; doi:10.1016/j.heliyon.2023.e18208)
Supplement: Multimedia component 1 [file mmc1.docx]

**Trends in the management of organic swine farm waste by composting: A systematic review**

Adriana Matiz-Villamil ^1*^, Kelly Johana Méndez-Carranza^2^, Andrés Felipe Pascagaza-Pulido^2^, Tatiana Rendón-Rendón^2^, Juliana Noriega-Noriega^2^_,_ Adriana Pulido-Villamarín ^2^

^1^ Laboratorio de Biotecnología Aplicada, Grupo de Biotecnología Ambiental e Industrial (GBAI), Departamento de Microbiología, Facultad de Ciencias, Pontificia Universidad Javeriana, Bogotá D.C., Colombia.

^2^ Unidad de Investigaciones Agropecuarias (UNIDIA), Departamento de Microbiología, Facultad de Ciencias, Pontificia Universidad Javeriana, Bogotá D.C., Colombia.

**Corresponding author***

**Adriana Matiz-Villamil,** M.Sc.

Associate Professor

Pontificia Universidad Javeriana, Bogotá, D.C., Colombia

Facultad de Ciencias

Departamento de Microbiología

Grupo de Biotecnología Ambiental e Industrial (GBAI)

Laboratorio de Biotecnología Aplicada

Postal Code: 110-23

E-mail: [amatiz@javeriana.edu.co](mailto:amatiz@javeriana.edu.co)

ORCID: [https://orcid.org/0000-0001-6528-4882](https://www-scopus-com.ezproxy.javeriana.edu.co/redirect.uri?url=https://orcid.org/0000-0001-6528-4882&authorId=12645150600&origin=AuthorProfile&orcId=0000-0001-6528-4882&category=orcidLink)

**Adriana Pulido-Villamarín,** M.Sc.

Assistant Professor

Pontificia Universidad Javeriana, Bogotá, D.C., Colombia

Facultad de Ciencias

Departamento de Microbiología

Unidad de Investigaciones Agropecuarias (UNIDIA)

Postal Code: 110-23

E-mail: [adriana.pulido@javeriana.edu.co](mailto:adriana.pulido@javeriana.edu.co)

ORCID: [https://orcid.org/0000-0002-1854-7886](https://www-scopus-com.ezproxy.javeriana.edu.co/redirect.uri?url=https://orcid.org/0000-0002-1854-7886&authorId=55102775900&origin=AuthorProfile&orcId=0000-0002-1854-7886&category=orcidLink)

Table S1. Articles included in the systematic review and their classification in the different categories. Microbial communities 3. Antibiotic resistance 4. Heavy metals 5. Polycyclic aromatic hydrocarbons 6. Parasitology 8. Phytopathogens 9. Nitrogen transformation 10. Bioinoculants 11. Comparison/combination with other waste management techniques 12. Factors affecting composting 13. Plant growth promotion/phytotoxicity.

| **References** | **Categories** | | | | | | | | | | | | | |
| --- | --- | --- | --- | --- | --- | --- | --- | --- | --- | --- | --- | --- | --- | --- |
|  | **1** | **2** | **3** | **4** | **5** | **6** | **7** | **8** | **9** | **10** | **11** | **12** | **13** | **14** |
| Yang *et al.* (2019) | X | X | X |  |  |  |  |  |  |  |  |  |  |  |
| de Sá *et al.* (2017) |  |  |  |  |  | X | X |  |  |  |  |  |  |  |
| Wolna-Maruwka *et al.* (2017) |  |  |  |  |  | X |  |  |  | X |  |  |  |  |
| Wong *et al.* (2016) | X |  |  |  |  | X |  |  |  |  |  |  |  |  |
| Ma *et al.* (2021) |  | X |  | X |  |  |  |  |  |  | X |  |  |  |
| Wang *et al.* (2021) | X | X |  | X |  |  |  |  |  |  |  |  |  |  |
| Arias *et al.* (2019) | X |  |  |  |  | X |  |  |  |  |  | X |  |  |
| Meng *et al.* (2018) | X |  |  |  |  |  |  | X |  |  |  |  |  | X |
| Yang e*t al.* (2019) |  | X |  |  |  |  |  |  | X | X |  |  | X |  |
| Meng *et al.* (2017) |  |  | X |  |  |  |  |  |  |  | X |  |  |  |
| Chi *et al.* (2020) |  | X |  |  |  |  |  |  |  | X |  |  |  |  |
| Ramires *et al.* (2019) |  |  |  |  |  | X | X |  |  |  | X |  |  | X |
| Yang *et al.* (2019) | X | X |  |  |  |  |  |  | X |  |  |  |  |  |
| Ren *et al.* (2020) | X |  |  |  |  |  |  |  | X |  |  | X |  |  |
| Wu *et al.* (2019) | X |  |  |  |  |  |  |  |  |  |  |  |  |  |
| Wang *et al*. (2020) | X |  | X |  |  |  |  |  |  |  |  |  |  |  |
| Meng et al. (2018) | X | X |  |  |  |  |  |  |  |  |  |  |  |  |
| Zhou (2018) | X |  |  |  |  |  |  |  |  |  |  | X |  |  |
| Liu *et al.* (2018) |  | X |  | X |  |  |  |  | X |  |  |  |  |  |
| Wu *et al.* (2017) | X |  | X |  |  |  |  |  |  |  |  |  |  |  |
| Lu *et al.* (2018) | X | X |  | X |  |  |  |  |  |  |  |  |  |  |
| Fan e*t al. (*2018) |  |  |  |  |  |  |  |  |  |  |  | X |  |  |
| Gao *et al.* (2019) | X |  |  | X |  |  |  |  |  |  |  |  |  |  |
| Jiang *et al.* (2018) | X |  |  |  |  |  |  |  | X |  |  |  |  |  |
| Wang *et al. (*2020) |  |  |  |  |  |  |  |  |  |  | X |  |  |  |
| Zhu *et al.* (2021) |  |  |  |  |  |  |  |  |  | X |  |  |  |  |
| Cheng *et al.* (2020) |  |  |  | X |  |  |  |  |  |  |  |  |  |  |
| Liu *al.* (2020) | X |  |  |  |  |  |  |  |  |  |  |  |  |  |
| Das *et al.* (2022) |  |  |  |  |  |  |  |  |  |  | X |  |  |  |
| Zhu *et al.* (2020) | X | X |  |  |  |  |  |  |  |  |  |  |  |  |
| Peng *et al.* (2020) |  | X |  | X |  |  |  |  |  |  |  |  |  |  |
| Wang *et al.* (*2020)* |  | X |  |  |  |  |  |  |  |  |  |  |  |  |
| Wan *et al.* (2021) |  | X |  |  |  |  |  |  |  |  |  |  |  |  |
| Matiz-Villamil *et al.* (2021) |  |  |  |  |  | X | X |  |  | X |  |  | X |  |
| Awasthi *et al.* (2020) |  |  |  |  |  | X |  |  |  |  | X |  |  |  |
| Ubani & Atagana (2018) |  |  |  |  | X |  |  |  |  |  |  |  |  |  |
| Chen *et al. (*2020) |  | X |  |  |  |  |  |  | X |  |  |  |  |  |
| Shehata *et al. (*2021) |  | X | X | X |  |  |  |  |  |  |  |  |  |  |
| Li *et al.* (2019) |  | X |  |  |  |  |  |  |  | X |  |  |  |  |
| Ma *et al.* (2020) |  | X |  | X |  |  |  |  |  |  |  |  |  |  |
| Zheng *et al.* (2020) |  |  |  |  |  |  |  |  | X |  |  |  |  |  |
| Vázquez, *et al. (*2017) |  |  | X |  |  |  |  |  | X |  |  |  |  |  |
| Liu & Wang (2017) |  |  |  |  |  |  |  |  | X | X |  |  |  |  |
| Gao *et al.* (2020) | X |  |  |  |  |  |  |  |  |  |  |  |  |  |
| Liu *et al.* (2018) | X |  |  |  |  |  |  |  |  |  |  |  |  |  |
| Galliou *et al.* (2018) |  |  |  |  |  |  |  |  |  |  | X |  |  |  |
| Chang *et al.* (2017) | X |  |  |  |  |  |  |  |  |  |  |  |  |  |
| Feng *et al.* (2019) |  |  |  |  |  |  |  |  | X |  |  |  |  |  |
| Li *et al. (*2019) | X |  | X |  |  |  |  |  |  | X |  |  |  |  |
| Li *et al.* (2019) | X | X |  |  |  |  |  |  |  |  |  |  |  |  |
| Duan *et al.* (2020) |  | X |  |  |  | X |  |  |  |  |  |  |  |  |
| Wang *et al. (*2018) |  | X |  |  |  |  |  |  |  |  |  |  |  |  |
| Tu *et al.* (2019) | X |  |  |  |  |  |  |  |  | X |  |  |  |  |
| Costa *et al. (*2021) |  |  |  |  |  |  |  |  |  |  |  |  | X |  |
| Bustamante *et al.* (2021) |  |  |  |  |  |  |  |  |  |  |  | X |  |  |
| Vargas-Sánchez *et al.* (2020) |  |  |  |  |  |  |  |  |  | X |  |  | X |  |
